# The Brief Case: Recalcitrant cutaneous and subcutaneous knee infection caused by *Purpureocillium lilacinum*

**DOI:** 10.1128/jcm.00448-26

**Published:** 2026-09-09

**Authors:** Ming-Chi Li, Tak-Wah Wong, Hsin-I Shih, Ming-I Hsieh, Wan-Lin Wu, Shin-Chen Pan, Chi-Jung Wu

**Affiliations:** 1 Division of Infectious Diseases, Department of Internal Medicine, National Cheng Kung University Hospital, College of Medicine, National Cheng Kung University 63461, Tainan, Taiwan; 2 Department of Dermatology, National Cheng Kung University Hospital, College of Medicine, National Cheng Kung University 63461, Tainan, Taiwan; 3 Department of Biochemistry and Molecular Biology, College of Medicine, National Cheng Kung University, Tainan, Taiwan; 4 Department of Emergency Medicine, National Cheng Kung University Hospital, College of Medicine, National Cheng Kung University 63461, Tainan, Taiwan; 5 National Institute of Infectious Diseases and Vaccinology, National Health Research Institutes 50115, Tainan, Taiwan; 6 Department of Surgery, Section of Plastic and Reconstructive Surgery, National Cheng Kung University Hospital, College of Medicine, National Cheng Kung University 63461, Tainan, Taiwan; Mayo Clinic Minnesota, Rochester, Minnesota, USA

**Keywords:** antifungal susceptibility testing, *Purpureocillium lilacinum*, split-thickness skin graft, therapeutic drug monitoring

## CASE

**Fig 1:**
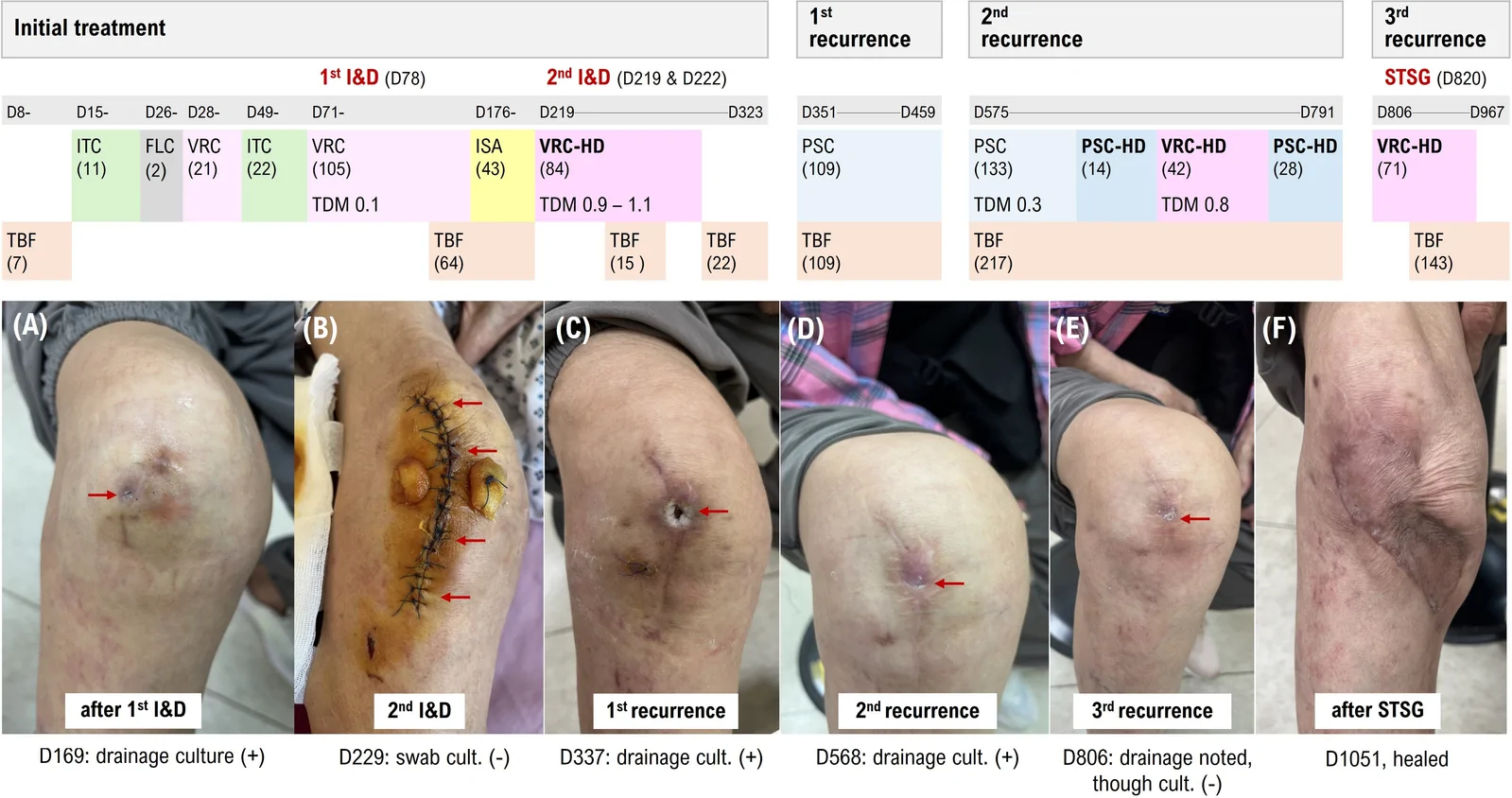
Timeline of the patient’s clinical course and antifungal therapy. Panels show (**A**) after the first I&D, (**B**) the second I&D, (**C**) the first recurrence, (**D**) the second recurrence, (**E**) the third recurrence, and (**F**) after STSG. Dx indicates the number of days after day 0, defined as the date of hospital presentation and first isolation of *P. lilacinum*. Numbers in parentheses indicate treatment duration in days. Culture (+) indicates growth of *P. lilacinum*, and culture (−) indicates no fungal growth. Arrows indicate sites of swab collection for fungal culture. Abbreviations: FLC, fluconazole; HD, high dose; I&D, incision and debridement; ISA, isavuconazole; ITC, itraconazole; PSC, posaconazole; STSG, split-thickness skin graft; TBF, terbinafine; TDM, therapeutic drug monitoring; VRC, voriconazole.

A 70-year-old woman with well-controlled diabetes mellitus (HbA1c, approximately 7.0%) presented to the hospital on day 0 (D0) with a nonhealing right knee wound accompanied by painful erythematous-to-purpuric swelling and serous drainage, 2 months after an accidental fall in an urban area. Terbinafine (250 mg/day) was initiated on D8 after a wound culture obtained on D0 yielded a filamentous fungus, designated isolate NCK1824. A lesional biopsy of the right knee wound performed on D8 also yielded an identical filamentous fungus. Both isolates were initially identified as *Paecilomyces* spp. based on morphology. Oral itraconazole (200 mg/day) was initiated on D15 and continued for 10 days, followed by sequential treatment with voriconazole (400 mg/day) for 3 weeks, itraconazole for 3 weeks, and reinitiation of voriconazole. Despite therapy, the lesion failed to heal, and repeat cultures continued to yield the same filamentous fungus. The first surgical debridement was performed on D78. The overall management course is summarized in Fig. 1 and Table S1.

**Fig 2:**
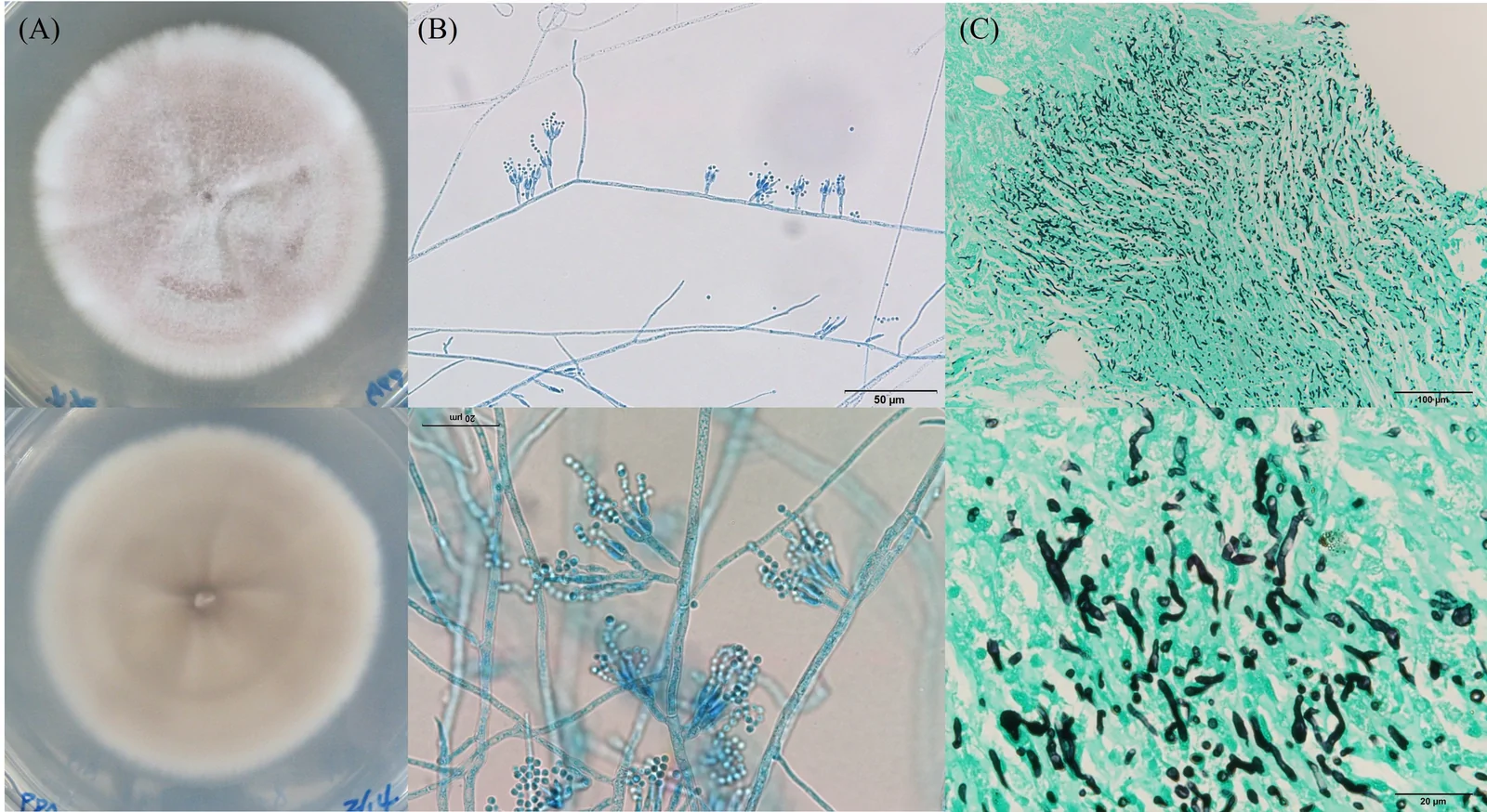
Mycological and histopathological features of *P. lilacinum* infection in this patient. (**A**) Floccose, conidiating lilac colony on potato dextrose agar after 10 days of incubation at 28°C (isolate NCK1824; upper, obverse; lower, reverse). (**B**) Micromorphology (lactophenol cotton blue stain) of isolates grown for 3 days at 28°C on Sabouraud dextrose agar: hyaline, septate hyphae with erect conidiophores bearing clusters of phialides. The phialides are swollen at the base and taper to slender necks, producing conidia in basipetal chains (upper, NCK1824, ×400; lower, NCK2152, ×1,000). (**C**) Histopathology of right knee subcutaneous tissue obtained at the second surgical debridement (D219), showing septate fungal hyphae on Grocott’s methenamine silver stain at low magnification (upper, ×200) and in a corresponding selected area at higher magnification (lower, ×1,000).

Isolate NCK1824 and subsequent knee isolates were later identified as *Purpureocillium lilacinum* by matrix-assisted laser desorption ionization–time of flight mass spectrometry (MALDI-TOF MS; VITEK MS, bioMérieux; Knowledge Base v3.2) and internal transcribed spacer (ITS) sequencing. The ITS sequence of isolate NCK1824 (GenBank accession no. LC912938) showed 100% identity to *P. lilacinum* UWFP 853 (accession no. AY213668.1). This identification was concordant with the phenotypic findings (Fig. 2A and B) and absence of growth at 45°C. Antifungal susceptibility testing of isolate NCK1824 using the European Committee on Antimicrobial Susceptibility Testing (EUCAST) broth microdilution method showed high minimum inhibitory concentrations (MICs) for amphotericin B (AMB) and itraconazole (both >16 µg/mL), whereas the MICs for voriconazole, posaconazole, isavuconazole, and terbinafine were 0.25, 0.5, 0.5, and 1 μg/mL, respectively (1). Voriconazole was continued, including a 12-day intravenous course; however, the wound remained nonhealing, and *P. lilacinum* continued to be isolated, even during combination therapy with voriconazole and terbinafine (Fig. 1A). Therapeutic drug monitoring (TDM) on D162 showed a subtherapeutic voriconazole trough concentration of 0.1 μg/mL. Although no organism-specific voriconazole trough target has been established for *P. lilacinum*, this concentration was well below the commonly used therapeutic range of 1.0 to 5.5 μg/mL extrapolated from invasive aspergillosis treatment guidance (2). Treatment was switched to isavuconazole for 6 weeks; however, wound cultures remained positive.

The patient subsequently underwent a second extensive debridement on D219 and D222 (Fig. 1B). Histopathology demonstrated numerous septate fungal hyphae (Fig. 2C), and cultures again yielded *P. lilacinum*. High-dose voriconazole was initiated at 600 mg/day on D219 and increased to 800 mg/day on D239, with corresponding trough concentrations of 0.9 and 1.1 μg/mL, which approximated the lower limit of the target range. Weekly wound cultures became negative after debridement, and the wound gradually healed. High-dose voriconazole was administered for a total of 84 days, followed by 21 days of terbinafine (250 mg/day) maintenance therapy. However, serous drainage from the knee recurred 2 weeks after terbinafine discontinuation, and cultures again yielded *P. lilacinum*, representing the first recurrence (Fig. 1C).

Standard-dose posaconazole (300 mg/day) plus terbinafine was administered for 109 days starting on D351, resulting in gradual wound healing. However, 4 months after posaconazole discontinuation, serous drainage from the knee recurred on D568, and *P. lilacinum* was again recovered as isolate NCK2152 (Fig. 1D), representing a second recurrence. Although wound cultures became negative 1 week after posaconazole was restarted on D575, ultrasonography on D679 suggested microabscesses. The posaconazole trough concentration was subtherapeutic at 0.3 μg/mL on D698, well below commonly used trough targets of >1 µg/mL for invasive aspergillosis and mucormycosis (2, 3). High-dose posaconazole (600 mg/day) was then administered beginning on D708, followed by sequential high-dose voriconazole and posaconazole for a total treatment duration of 84 days, resulting in apparent wound healing. However, serous drainage from the knee recurred 2 weeks after treatment discontinuation, constituting a third recurrence (Fig. 1E).

Given the refractory course, the patient underwent wide excision with gastrocnemius muscle flap and split-thickness skin graft (STSG) reconstruction on D820. She received perioperative and postoperative high-dose voriconazole (800 mg/day) for 10 weeks (D806 to D876) in combination with terbinafine (500 mg/day, then 250 mg/day), followed by terbinafine maintenance monotherapy (500 mg/day, then 250 mg/day) for 92 days. The wound healed completely (Fig. 1F), and no relapse occurred during 1 year of follow-up after STSG.

Sequential *P. lilacinum* isolates had identical ITS sequences and similar antifungal susceptibility profiles, supporting relapsing infection (Table S2). Throughout the clinical course, 17 aerobic/anaerobic bacterial cultures and 5 mycobacterial cultures from drainage or tissue specimens obtained from the right knee wound were negative. A distinct episode of a 1 cm, culture-confirmed, superficial *Pseudomonas aeruginosa* abscess developed at the right knee wound on D721 and resolved after 7 days of oral ciprofloxacin. Magnetic resonance imaging showed no evidence of joint involvement or osteomyelitis. *CYP2C19* genotyping revealed a CYP2C19 *1/*1 genotype, indicating a normal voriconazole metabolizer phenotype (4).

## DISCUSSION

*P. lilacinum* is a ubiquitous, saprophytic, hyaline ascomycetous mold with worldwide distribution. It has been isolated from soil, air, decaying vegetation, insects, and nematodes; can colonize synthetic materials, including medical devices such as catheters, plastic implants, and saline bottles; and can persist on moist surfaces in water distribution systems (5, 6). It is increasingly recognized as an opportunistic cause of hyalohyphomycosis in both immunocompromised and immunocompetent hosts (7). The most frequently reported clinical manifestations are ocular infections, including keratitis and endophthalmitis, and cutaneous infections (7–9). Traumatic inoculation, as in the present case, and iatrogenic inoculation, as illustrated by a recent outbreak of *P. lilacinum* keratitis, are recognized routes of entry for localized infection (7, 8, 10). Clinical manifestations of cutaneous or subcutaneous *P. lilacinum* infection vary and include erythematous macules, papules, vesicles, nodules, abscesses, and cellulitis; spontaneous resolution is uncommon (8, 11). Optimal management usually requires prolonged antifungal therapy, often for at least 12 weeks, combined with surgical intervention (7). We present a recalcitrant case of cutaneous and subcutaneous knee infection caused by *P. lilacinum* in an immunocompetent patient, characterized by three recurrences despite prolonged antifungal therapy and repeated surgical debridement. Definitive cure was achieved after wide excision with muscle flap and STSG reconstruction, combined with high-dose voriconazole plus terbinafine therapy. This case highlights five key elements for the successful management of refractory *P. lilacinum* infection.

First, fungal infection should be considered in chronic, nonhealing wounds that respond poorly to antibacterial therapy, and fungal cultures should be obtained from affected lesions to establish an etiologic diagnosis.

Second, accurate species identification is essential. *Purpureocillium lilacinum* was formerly classified as *Paecilomyces lilacinus* based on morphology (5). However, phylogenetic analyses of 18S rRNA, ITS, and TEF1-α sequences showed that it is distinct from *Paecilomyces sensu stricto*, represented by *Paecilomyces variotii*, leading to its reclassification in the genus *Purpureocillium* as *Purpureocillium lilacinum* (5). *Purpureocillium lilacinum* can be distinguished from the thermotolerant species *Paecilomyces variotii* by ITS or D1/D2 sequence analysis, by MALDI-TOF MS when validated reference spectra are available, and by its lack of growth at 45°C (5, 7, 12). This distinction is crucial for patient management because the two species differ in antifungal susceptibility profiles and clinical responses to antifungal agents (7). In general, *P. variotii* shows lower MICs for amphotericin B and itraconazole, often <0.5 µg/mL, whereas *P. lilacinum* shows higher MICs for these agents; in contrast, *P. lilacinum* shows lower MICs for voriconazole, often <0.5 µg/mL, whereas *P. variotii* shows higher MICs (12, 13). In line with these susceptibility profiles, global guidelines recommend liposomal AMB (L-AMB) as first-line therapy for *Paecilomyces* infection and voriconazole for *Purpureocillium* infection, with a limited role for voriconazole in *Paecilomyces* infection (7).

Third, antifungal susceptibility testing may inform treatment strategies. Voriconazole plus terbinafine is recommended as first-line therapy for cutaneous or subcutaneous *Purpureocillium* infection, given terbinafine’s high affinity for skin and adipose tissue, and itraconazole, L-AMB, and posaconazole are considered second-line options (7). At present, MIC interpretation for *P. lilacinum* remains limited by the absence of established epidemiologic cutoff values and clinical breakpoints that correlate MICs with clinical outcomes. However, MIC data may still provide supportive information to inform antifungal therapy. In our case, serial isolates consistently showed high MICs for itraconazole and AMB (>8 µg/mL), which, together with published reports of treatment failure with these agents (14–17), made them less favorable therapeutic options.

Voriconazole and posaconazole have been used successfully in several reported cases of cutaneous *P. lilacinum* infection, either alone or in combination with surgical intervention (7, 11, 14, 15, 18–20). Using the EUCAST method, one study of 27 *P. lilacinum* isolates reported MIC50/90 values of 0.5/1 µg/mL for voriconazole and 0.25/0.5 µg/mL for posaconazole, whereas another study of 28 isolates reported MIC50/90 values of 0.25/0.5 µg/mL for both agents (12, 13). In our case, the MICs for voriconazole and posaconazole were 0.25 and 0.5 µg/mL, respectively, within the ranges reported in these studies. Together with prior reports of favorable clinical outcomes, these findings supported the use of voriconazole and posaconazole in our patient.

Fourth, adequate antifungal exposure should be considered. In our case, recurrence later developed despite high-dose voriconazole combined with surgical debridement, with a voriconazole trough concentration of 1.1 µg/mL, and again after standard-dose posaconazole therapy, with a posaconazole trough concentration of 0.3 µg/mL. These findings suggest that higher voriconazole and posaconazole exposure may be needed to achieve sustained control of cutaneous *P. lilacinum* infection. To date, optimal therapeutic serum concentration targets for *P. lilacinum* infection remain undefined. Further studies are needed to establish clinical breakpoints and therapeutic concentration targets for this organism.

Voriconazole is metabolized predominantly by CYP2C19 and exhibits substantial interpatient variability in serum concentrations, partly attributable to CYP2C19 genetic polymorphisms, whereas isavuconazole and posaconazole are less dependent on CYP2C19 metabolism (4). The subtherapeutic voriconazole concentration raised concern that the patient might have a rapid or ultrarapid metabolizer phenotype. However, CYP2C19 genotyping revealed a normal metabolizer phenotype. In addition, the posaconazole concentration was also subtherapeutic at standard dosing, and cultures remained positive during isavuconazole therapy. These findings suggest that factors beyond CYP2C19 status may have contributed to the apparently low triazole exposure in this patient.

Finally, definitive surgical source control may be necessary for recurrent disease, as recurrence of cutaneous or subcutaneous *P. lilacinum* infection after an apparent clinical response has been documented. One immunocompetent patient developed recurrent infection 8 years after completing 10 weeks of voriconazole plus surgical excision; sustained cure was achieved with 6 months of posaconazole followed by excision of the residual lesion (21). A farmer with Evans syndrome receiving prednisolone relapsed 5 months after 12 weeks of voriconazole and was ultimately cured with surgical debridement, STSG, and 11 weeks of posaconazole (22). A renal transplant recipient relapsed 1 week after completing 6 months of posaconazole therapy, with recurrence attributed in part to a retained splinter; the infection resolved after complete extraction of the foreign body and 9 months of posaconazole therapy (23). Together, these observations suggest that *P. lilacinum* can persist in cutaneous tissue despite apparent clinical resolution, regardless of host immune status. Therefore, in recurrent cases, particularly when azole exposure is inadequate or the patient is immunocompromised, definitive surgery, such as muscle flap and STSG reconstruction, may be necessary to achieve durable remission. Well-vascularized muscle may enhance infection control by increasing blood flow, immune cell delivery, and local exposure to systemic antifungal agents.

In conclusion, this case of recalcitrant knee *P. lilacinum* infection underscores key management considerations, including maintaining suspicion for fungal infection in chronic nonhealing wounds, ensuring accurate species identification, performing antifungal susceptibility testing, using TDM to inform antifungal therapy, and implementing definitive surgical source control, particularly in recurrent disease

## SELF-ASSESSMENT QUESTIONS

1. Which of the following statements best describes cutaneous/subcutaneous *Purpureocillium lilacinum* infection?
  - *Paecilomyces* species and *P. lilacinum* share similar antifungal susceptibility profiles.
  - It is prone to spontaneous resolution without recurrence.
  - Amphotericin B is recommended as first-line therapy.
  - Voriconazole plus terbinafine is recommended as first-line therapy.
2. Which of the following statements is false regarding the diagnosis and species identification of *P. lilacinum* infection?
  - Fungal culture from the affected site, followed by species identification of the recovered isolate, is recommended.
  - Morphological characteristics alone are sufficient for species identification.
  - Sequence analysis of the ITS or 28S rDNA D1/D2 regions can aid species identification.
  - MALDI-TOF MS can be used for species identification when validated reference spectra are available.
3. Which of the following is least appropriate for managing refractory or recurrent cutaneous/subcutaneous *P. lilacinum* infection?
  - Antifungal susceptibility testing to help inform antifungal therapy.
  - Therapeutic drug monitoring to identify inadequate azole exposure.
  - Antifungal therapy alone without surgical intervention.
  - Definitive surgical source control in cases with inadequate drug exposure.

## ANSWERS TO SELF-ASSESSMENT QUESTIONS

1. Which of the following statements best describes cutaneous/subcutaneous *Purpureocillium lilacinum* infection?
  - *Paecilomyces* species and *P. lilacinum* share similar antifungal susceptibility profiles.
  - It is prone to spontaneous resolution without recurrence.
  - Amphotericin B is recommended as first-line therapy.
  - Voriconazole plus terbinafine is recommended as first-line therapy.

Answer: d. *P. lilacinum* should be distinguished from *Paecilomyces* species because their antifungal susceptibility profiles differ. Cutaneous/subcutaneous *P. lilacinum* infection usually does not resolve spontaneously and may recur. According to the global guideline, voriconazole plus terbinafine is recommended as first-line therapy for cutaneous/subcutaneous *Purpureocillium* infection.

1. Which of the following statements is false regarding the diagnosis and species identification of *P. lilacinum* infection?
  - Fungal culture from the affected site, followed by species identification of the recovered isolate, is recommended.
  - Morphological characteristics alone are sufficient for species identification.
  - Sequence analysis of the ITS or 28S rDNA D1/D2 regions can aid species identification.
  - MALDI-TOF MS can be used for species identification when validated reference spectra are available.

Answer: b. Microbiological differentiation between *Purpureocillium lilacinum* and morphologically similar *Paecilomyces* species is challenging. Accurate identification can be supported by sequence analysis of the ITS or 28S rDNA D1/D2 regions, and MALDI-TOF MS may also assist when validated reference spectra are available.

1. Which of the following is least appropriate for managing refractory or recurrent cutaneous/subcutaneous *P. lilacinum* infection?
  - Antifungal susceptibility testing to help inform antifungal therapy.
  - Therapeutic drug monitoring to identify inadequate azole exposure.
  - Antifungal therapy alone without surgical intervention.
  - Definitive surgical source control in cases with inadequate drug exposure.

Answer: c. Although *P. lilacinum* often demonstrates favorable *in vitro* susceptibility to voriconazole and reduced susceptibility to amphotericin B and itraconazole, susceptibility may vary among isolates; therefore, antifungal susceptibility testing can help inform antifungal therapy. Therapeutic drug monitoring is also useful for identifying inadequate azole exposure. Because *P. lilacinum* infection may persist in cutaneous or subcutaneous tissue despite apparent clinical improvement, antifungal therapy alone may be insufficient in refractory or recurrent localized disease, and definitive surgical source control should be considered.

**Take-Home Points**

1. *Purpureocillium lilacinum* is a ubiquitous environmental mold that can cause ocular and cutaneous/subcutaneous infections, often after traumatic inoculation.
2. Compared with *Paecilomyces variotii*, *P. lilacinum* typically shows lower MICs for voriconazole but higher MICs for amphotericin B and itraconazole.
3. Voriconazole is recommended as first-line therapy for *Purpureocillium* infection.
4. Cutaneous/subcutaneous *P. lilacinum* infection may recur, and antifungal susceptibility testing and therapeutic drug monitoring may help inform antifungal therapy.
5. For refractory or recurrent disease, particularly when antifungal exposure is suboptimal, definitive surgical source control, such as wide excision with split-thickness skin graft reconstruction, may be necessary.

## Data Availability

The data supporting the findings of this study are available within the paper and its supplemental material.

## SUPPLEMENTAL MATERIAL

Tables S1 and S2 jcm.00448-26-s0001.docx Table S1: Timeline of clinical course. Table S2: Antifungal susceptibility profiles.

ASM does not own the copyrights to Supplemental Material that may be linked to, or accessed through, an article. The authors have granted ASM a non-exclusive, world-wide license to publish the Supplemental Material files. Please contact the corresponding author directly for reuse.
