# Supplementary material for: The Brief Case: Recalcitrant cutaneous and subcutaneous knee infection caused by Purpureocillium lilacinum
Source: J Clin Microbiol. 2026 Sep 9;64(9):e00448-26. doi: 10.1128/jcm.00448-26 (PMC13556207; doi:10.1128/jcm.00448-26)
Supplement: Supplementary file 1 [file jcm.00448-26-s0001.docx]

**Table S1**. Timeline (dates) of clinical course, management, and culture results

| Antifungals and dose | Administration date^#^ | Culture positive for *P. lilacinum* | TDM | Surgery |
| --- | --- | --- | --- | --- |
| Initial treatment |  |  |  |  |
| TBF 250 mg qd | D8 – D14 | D0, D8 |  |  |
| ITC 100 mg q12h | D15 – D25 |  |  |  |
| FLC 400 mg qd | D26 – D27 (iv) |  |  |  |
| VRC 200 mg q12h | D28 – D34 (iv) | D28 |  |  |
|  | D35 – D48 |  |  |  |
| ITC 100 mg q12h | D49 – D70 | D64 |  |  |
| VRC 200 mg q12h | D71 – D74 | D71 |  |  |
|  | D75 – D86 (iv) | D78, D86 |  | 1^st^ I&D (D78): |
|  | D87 – D175 | D106, D155, D169, D176 | VRC 0.1 (D162) |  |
| ISA 200 mg qd^&^ | D176 – D218 | D183, D189, D197, D204 |  |  |
| TBF 250 mg qd | D155 – D218 |  |  |  |
| VRC 200 mg q8h^&^ | D219 – D230 | D219 | VRC 0.9 (D225) | 2^nd^ I&D (D219, 222) |
|  | D231 – D238 (iv) |  |  |  |
| VRC 200 mg q6h | D239 – D249 (iv) |  | VRC 1.1 (D246) |  |
|  | D250 – D287 |  |  |  |
| TBF 250 mg qd | D260 – D274 |  |  |  |
| VRC 200 mg q8h | D288 – D302 |  |  |  |
| TBF 250 mg qd | D303 – D323 |  |  |  |
| 1^st^ recurrence, culture-proven |  | D337 |  |  |
| PSC 300 mg qd^&^ | D351 – D459 |  |  |  |
| TBF 250mg qd | D351 – D459 |  |  |  |
| 2^nd^ Recurrence, culture-proven |  | D568 |  |  |
| PSC 300 mg qd | D575 – D707 |  | PSC 0.3 (D698) |  |
| PSC 300 mg q12h | D708 – D721 |  |  |  |
| VRC 200 mg q6h | D722 – D749 |  |  |  |
| VRC 200 mg q8h | D750 – D763 |  |  |  |
| PSC 200 mg q12h | D764 – D791 |  |  |  |
| TBF 250mg qd | D575 – D791 |  |  |  |
| 3^rd^ recurrence, clinical suspicion | |  |  |  |
| VRC 200mg q6h | D806 – D819 |  | VRC 0.8 (D813) |  |
|  | D820 – D824 (iv) |  |  | STSG (D820) |
| VRC 400 mg q12h | D825 – D876 |  |  |  |
| TBF 250 mg q12h | D825 – D862 |  |  |  |
| TBF 250 mg qd | D863 – D876 |  |  |  |
| TBF 250 mg q12h | D877 – D903 |  |  |  |
| TBF 250 mg qd | D904 – D967 |  |  |  |

^#^Antifungals were administered orally, except agents labeled as iv, which were given intravenously.

^&^Loading: ISC 200mg q8h for 3 doses; VRC 400mg q8h for 3 doses orally; PSC 300mg q12h for 2 doses

D*x* indicates the number of days after day 0, defined as the date of hospital presentation and first isolation of *P. lilacinum*. Abbreviation: FLC, fluconazole; I&D, incision and debridement; ISC, isavuconazole; ITC, itraconazole; PSC, posaconazole; STSG, split-thickness skin graft; TBF, terbinafine; VRC, voriconazole.

**Table S2.** Antifungal susceptibility profiles of serial *P. lilacinum* knee isolates determined using CLSI M38-A3, with EUCAST testing performed for the initial isolate.

| Antifungal agent | MIC or MEC (μg/mL) by isolate (collection date and strain no.) | | | | | | | |
| --- | --- | --- | --- | --- | --- | --- | --- | --- |
|  | D0 | D8 | D64 | D155 | D169 | D197 | D337 | D568 |
|  | NCK1824 | NCK1829 |  |  |  |  |  | NCK2152 |
| Method | EUCAST/  CLSI | CLSI | CLSI | CLSI | CLSI | CLSI | CLSI | CLSI |
| Amphotericin B | >16 / >8 | >8 | >8 | >8 | >8 | >8 | >8 | >8 |
| Itraconazole | >16 / >16 | >16 | >16 | >16 | >16 | >16 | >16 | 16 |
| Voriconazole | 0.25 / 0.5 | 0.5 | 0.5 | 0.5 | 0.5 | 0.5 | 0.25 | 0.25 |
| Posaconazole | 0.5 / 0.5 | 0.5 | 0.5 | 0.5 | 0.5 | 0.5 | 0.25 | 0.25 |
| Isavuconazole | 0.5 / 0.5 | 0.5 | 0.5 | 0.5 | 0.5 | 0.5 | 0.5 | 0.25 |
| Terbinafine | 1 / 1 | 1 | 1 | 1 | 1 | 1 | 0.5 | 1 |
| Ketoconazole | − / 1 | 1 | 1 | − | − | 1 | 1 | 1 |
| Anidulafungin | − / <0.12 | <0.12 | <0.12 | − | − | <0.12 | − | − |
| Flucytosine | − / >64 | >64 | >64 | − | − | >64 | − | − |

Abbreviation: MECs, minimum effective concentrations (for anidulafungin); MICs, minimum inhibitory concentrations (for all other antifungal agents);－, not performed.
